# Swim Bladder Disorders in Koi Carp (*Cyprinus carpio*)

**DOI:** 10.3390/ani10111974

**Published:** 2020-10-28

**Authors:** Rubina Sirri, Luciana Mandrioli, Samuele Zamparo, Francesca Errani, Enrico Volpe, Giorgia Tura, Tim Barbé, Sara Ciulli

**Affiliations:** 1 Department of Veterinary Medical Sciences—Alma Mater Studiorum University of Bologna, 40064 Bologna, Italy; rubina.sirri2@unibo.it (R.S.); samuniud@gmail.com (S.Z.); francesca.errani2@unibo.it (F.E.); enrico.volpe2@unibo.it (E.V.); giorgia.tura3@unibo.it (G.T.); sara.ciulli@unibo.it (S.C.); 2 DAP Tim Barbe and an der Steenstraat 45, 1750 Lennik (Vlaams-Brabant), Belgium; tim.barbe@gmail.com

**Keywords:** ornamental fish, koi carp, cyprinid, swim bladder disorders, diagnostic imaging, microbiology, histopathology

## Abstract

**Simple Summary:**

Physostomous fish like cyprinids possess a swim bladder with a pneumatic duct in connection with the foregut, permitting the gas to enter into or be released through the alimentary canal. Due to this peculiar anatomic structure, bacteria and mycetes can potentially ascend the duct and colonize the swim-bladder. Besides inflammatory reactions, other swim bladder disorders include fluid accumulation, collapse, overinflation, and herniation. These swim bladder disorders and consequent buoyancy problems can be encountered in ornamental fish. Nonetheless, details about comprehensive disease management are poorly represented in the koi carp (*Cyprinus carpio*) literature. A clinical–pathological and microbiological investigation was performed in nine koi carp presenting abdominal swelling and abnormal swimming behavior. Swim bladder deformation, accumulation of clear fluid within the swim bladder, chronic aerocystitis, and bacteria identified as Aeromonas hydrophila/caviae group and *Shewanella xiamenensis* were the main findings. A wide range of sensitivity was shown to antimicrobials for isolated *S. xiamemensis* strains. Accordingly, antibiotic treatment succeeded in the full recovery of three cases in which *S. xiamemensis* infection was detected. Along with these results, the contribution of this study to the scientific field is to document a multidisciplinary clinical–pathological and microbiological investigation of these animals, which are rightly considered pets and should be similarly approached.

**Abstract:**

Swim bladder disorders and consequent buoyancy problems are encountered in ornamental fish, including koi carp. Nevertheless, beyond clinical and pharmacological management, they are largely underdiagnosed. In this study, nine koi carp showing abdominal swelling and abnormal swimming behavior were investigated. Clinical approach, varying from case to case, included ultrasonographic and X-ray investigations, bacteriological analysis of the collected fluid, antimicrobial susceptibility pattern, and possibly histological analysis. Diagnostic imaging, corroborating gross examination, documented swim bladder deformation/dislocation and serous fluid within the swim bladder chambers of most animals. Bacteria belonging to the Aeromonas hydrophila/caviae group and *Shewanella xiamenensis* were identified. *S. xiamenensis* strains showed a sensibility to all tested molecules except for one strain, which was resistant to tetracycline and cyprofloxacin. Antibiotic treatment succeeded in the full recovery of three cases in which *S. xiamemensis* infection was detected. Chronic aerocystitis was histologically documented where tissue was available. The swim bladder histopathological findings highlighted a chronic process that had compromised the quality of life of the animals. A multidisciplinary clinical–pathological and microbiological approach is highly suggested to recognize swim bladder conditions as early as possible, aiming to drive medical intervention and raising the chances of fish survival.

## 1. Introduction

Koi carp (*Cyprinus carpio*), abbreviation of “Nishikigoi”, the original Japanese name, are ornamental species of great economical interest worldwide. The systematic breeding of colorful carp in Japan began in Niigata in the 1820s and the fascination and interest towards these fish started to spread throughout Japan and, as the hobby grew internationally, many farms sprung up all over the world driven by the need to meet hobbyists’ demand. Actually, there are more than 100 varieties of koi created through breeding, and their economic value depends on accredited characteristics such as body conformation, swimming style, color quality, color durability, and color distribution, raising thousands of dollars in value [1,2,3,4]. This growing interest in koi carp has also increased the interest of the scientific community in their health problems [5]. Health issue of interest comprehends both diffusive diseases with a high impact on large fish population [6] and disorders that impact on single subjects such as neoplasia [7]. Koi carp are actually considered as pet fish: this fish species has a long life span, a high economical value, and a high emotional value due to its tame character [2,8]. Thus, fish keepers take care of them, providing medical assistance when needed even for individual specimens, increasingly turning to the veterinary profession [2,4].

Swim bladder disorders and consequent buoyancy problems are encountered in ornamental fish species, especially goldfish (*Carassius auratus*) [9]. In the literature, there are very few publications describing this pathology [9,10]; on the other hand, swim bladder disorders are being increasingly observed according to breeders/importers and are tentatively managed in veterinary clinical practice. Several etiologies have been proposed as the cause of this condition, including poor water quality, infectious agents, poor nutrition, neoplasia, injuries, and genetic factors [9,10]. Disorders of the swim bladder include abnormal fluid accumulation, collapse, overinflation, and herniation. Typical clinical signs include abdominal swelling, abnormal swimming behavior, and the loss of neutral buoyancy, with the fish either sinking to the bottom or floating on the surface. The buoyancy problems are caused by a reduced gas volume and the presence of fluid within the swim bladder (commonly called “swim bladder flooding”), which is easily detectable using diagnostic imaging [2,10]. Recently, an outbreak of swim bladder inflammation (SBI) in koi carp in Taiwan was associated with the presence of *Sphaerospora dykovae* [11]. Indeed, SBI is a well-known acute severe disease of common carp (*Cyprinus carpio*) fingerlings, studied since the 1980s, associated with the myxozoan *Sphaerospora dykovae* (previously *S. renicola*) [12], which has a significant economic impact in Central Europe [13]. However, data on ornamental koi carp (*Cyprinus carpio koi*) swim bladder disorders and their etiology are lacking in the literature. The present work aimed to describe a comprehensive investigation of nine cases of swim bladder disorder in adult koi carp. 

## 2. Materials and Methods 

### 2.1. Animals and Sampling

Nine adult koi carp (total length 62 ± 14 cm) held in different private ponds in Belgium were presented from 2016 to 2019 to the veterinary practitioner co-author for a visible abdominal swelling, deformation, and abnormal swimming behavior characterized by loss of neutral buoyancy, floating on the surface, and laying on the bottom (Table 1). The swim bladder disorder diagnosis was made after a total clinical examination and the application of diagnostic techniques recommended for internal disorders of koi carp [2].

Particularly, typical history associated with described cases was: all other fish in the pond were looking healthy, ate properly, the water quality was fine, no fish dead in the previous couple of weeks, only one fish laid on the bottom of the pond showing difficulty to swim to the surface, using the cranial fins extensively, eating but having trouble to reach the food, parking on the bottom while not swimming and using the cranial fins to balance, and the ventral part of the abdomen of this fish generally presented bedsores.

Six koi were euthanized with a lethal dose of anesthetic (Tricaine methanesulfonate MS-222, Merck KGaA, Darmstadt, Germany), with the owners’ agreement, due to the worsening of their clinical condition (cases 1#–6#) and three koi successfully recovered after antibiotic treatment (cases 7#–9#). The clinical approach varied from case to case, considering the chances of survival and the intentions of the owner (Table 1): eight fish underwent ultrasonographic investigation and two fish underwent X-rays, seven fish out of nine underwent bacteriological investigations of aspirated swim bladder fluid, while all six euthanized fish underwent histology (Table 1).

### 2.2. Bacteriological Examination

Swim bladder fluid was collected in sterile tubes via fine-needle aspiration from a total of seven fish (four euthanized and three alive).

Swim bladder fluid was collected in alive fish though echo-guided fine-needle aspiration, whereas in euthanized fish, it was collected during autopsy from the swim bladder placed in sight, by needle aspiration. All procedures were conducted under sterile condition. The collected fluid was immediately cultured on Columbia blood agar at 28 °C for a minimum of 24 h. Pure cultures were then cultured on AD plates (ampicillin dextrin agar, Tritium Microbiologie, Eindhoven, the Netherlands). Identification was done phenotypically by growth on Kligler–Iron agar, oxidase test and API 20 NE (BioMèrieux, Schaerbeek, Belgium), and selection was based on morphology, growth, pigmentation, and odor. Four isolates were further characterized through rDNA 16S amplification and sequencing. Briefly, DNA was extracted from colonies via the boiling method, and amplification of 16S rDNA was performed with P0F and P6R primers using 0.1 mL of each lysed cell suspension according to the procedure described previously [14]. Polymerase chain reaction products were purified and sequenced to confirm their bacterial identity. Sequences were obtained through the Bio-Fab Sequencing Service (Rome, Italy) and then analyzed through the online Basic Local Alignment Search Tool (BLAST) web interface provided by the National Center for Biotechnology Information (NCBI) to confirm bacterial identity [15]. An antimicrobial susceptibility pattern test against tetracycline, cloramphenicol, sulfametaxone/trimethoprim, cyprofloxacin, ampicillin/sulbactam, and amikacin was performed on four samples using the Kirby–Bauer disk diffusion method on Mueller Hinton agar [16].

### 2.3. Histology

The entire swim bladders of six euthanized fish were sampled during necropsy and fixed in 10% buffered formalin for histological investigations. Samples were then processed for routine histology, and sections were cut at 4 µm and stained with hematoxylin–eosin (H&E) and periodic acid–Schiff (PAS). 

## 3. Results

### 3.1. Diagnostic Imaging and Gross Findings

**Figure 1:**
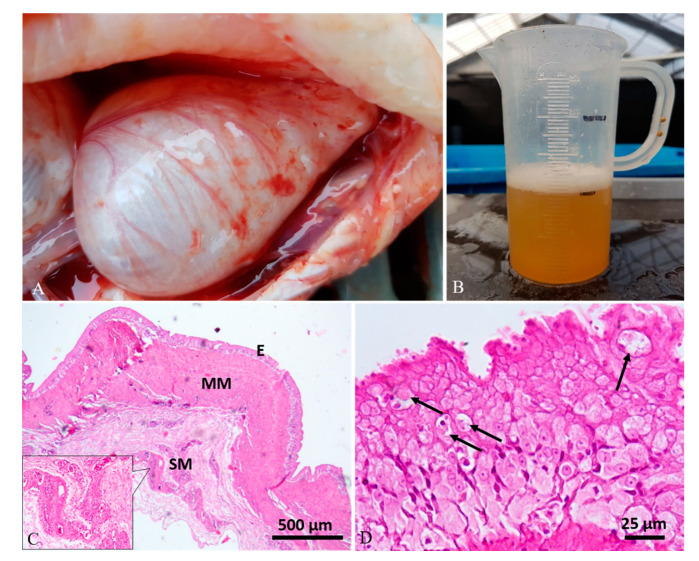
Case 4#. (**A**) Swim bladder showing a severe enlargement of chambers and multifocal superficial hyperemic streaks. (**B**) A conspicuous amount of yellow clear fluid was aspirated from the swim bladder. (**C**) Histological analysis showed severe thickening of the muscularis mucosae (MM) and perivascular inflammatory-cell infiltration in the submucosa (SM) (inset; hematoxylin–eosin (H&E) stain). (**D**) The mucosa (**E**) showed a severe epithelial hyperplasia with the presence of intracytoplasmic eosinophilic round aggregates compatible with parasitic stages (arrows) (H&E stain).

**Figure 2:**
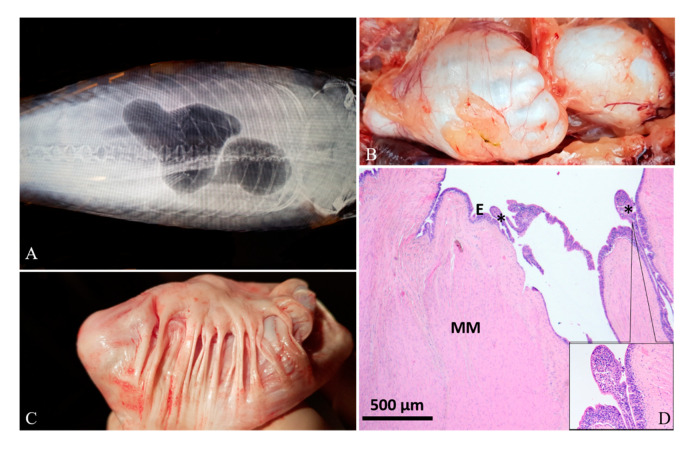
Case 5#. (**A**) Radiographic image of swim bladder showing abnormal shape. (**B**) Grossly, the chambers, especially the caudal one, showed an irregular surface. (**C**) At the opening, the internal surface was characterized by abundant fibrous connective tissue that twitched the swim bladder wall. (**D**) Histology shows a severe fibrous hyperplasia of the muscularis mucosae (MM) and a severe mucosal (**E**) hyperplasia forming papillae (asterisks), with abundant inflammatory cell exocytosis (inset; H&E stain).

**Figure 3:**
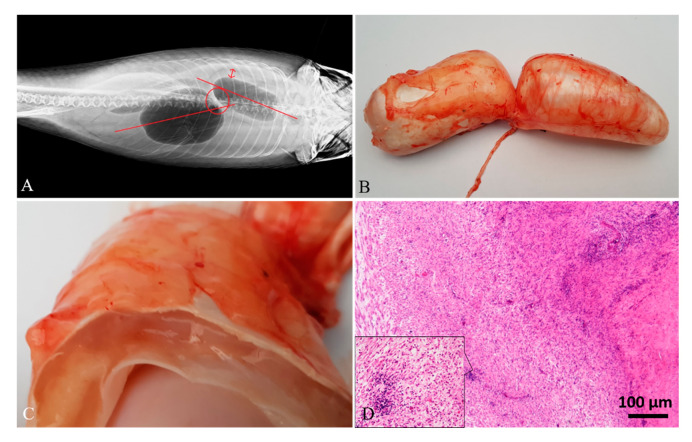
Case 6#. (**A**) Radiographic image of swim bladder showing dislocation of the chambers. (**B**) Grossly, the size of the chambers appeared slightly enlarged. (**C**) When cut in a transversal section, abundant gelatinous material expanding the swim bladder layers was detected. (**D**) Histological analysis showed the muscularis mucosae expanded by abundant granulation tissue and a severe inflammatory cell infiltration, mainly composed of lymphocytes (inset; H&E stain).

**Figure 4:**
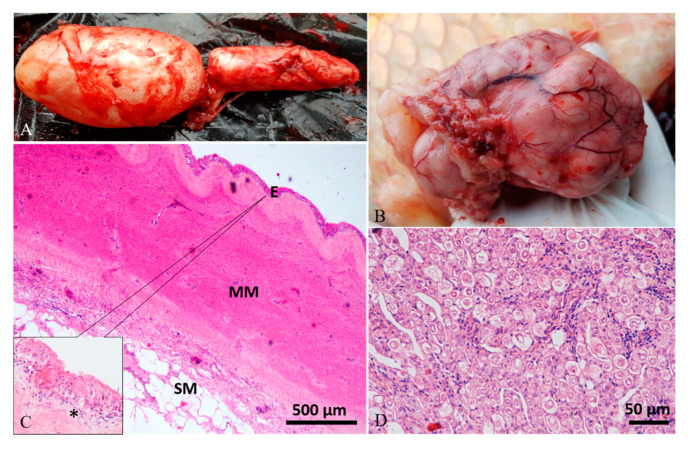
Case 1#. (**A**) Swim bladder showing a moderate enlargement of caudal chamber. (**B**) The fish had a large intracoelomic tumor, which caused the compression of the swim bladder. (**C**) Histological analysis of the swim bladder showed fibrous hyperplasia of the muscularis mucosae (MM), submucosal (SM) multifocal inflammatory cell infiltration, edema of lamina propria (asterisk in inset), and mucosal (**E**) hyperplasia with squamous metaplasia (inset; H&E stain). (**D**) Histology of the tumor revealed a gonadal germ-cell tumor (H&E stain).

Diagnostic imaging and autoptic examination revealed in six fish the presence of yellow clear fluid (serous exudate) within the swim bladder that caused enlargement of chambers. One of these fish (case 4#) also showed multifocal hyperemic streaks on the swim bladder’s external surface (Figure 1A,B). One fish (case 5#) presented a severe malformation of both swim bladder chambers with an irregular external surface, and the internal wall twitched in the presence of abundant fibrous connective tissue (Figure 2A–C). One fish (case 6#) presented a severe swim bladder misalignment and enlargement of chambers caused by the presence of abundant gelatinous material expanding the swim bladder layers detected in a transversal cut section (Figure 3A–C). One fish (case 1#) had a coelomic mass, histologically diagnosed as a gonadal germ-cell tumor, which compressed the swim bladder (Figure 4A,B,D), and one fish (case 3#) had supernumerary swim bladder.

### 3.2. Histology

Sub-grossly, the thickness of the swim bladder wall ranged from 0.7 to 7.7 mm in the six fish examined; in three fish (cases 1#, 5#, 6#), the swim bladder wall appeared to be markedly thickened. 

**Figure 5:**
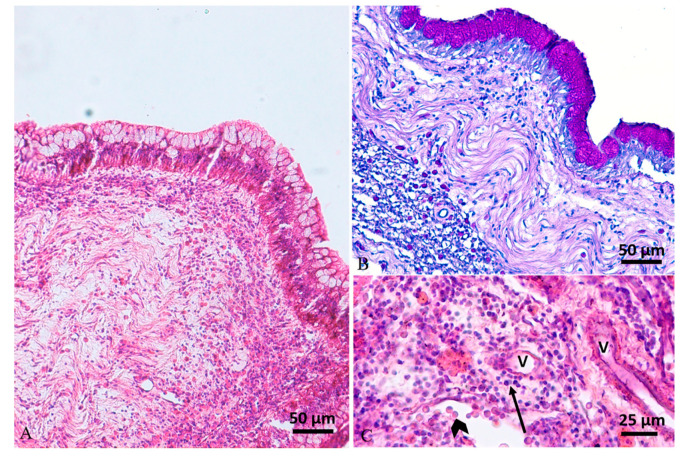
Case 2#. (**A**) Histology of swim bladder showing mucosal hyperplasia with mucous metaplasia and a severe mixed inflammatory cell infiltration expanding the lamina propria and muscularis mucosae (H&E stain). (**B**) Periodic acid–Schiff (PAS) stain highlights mucous metaplasia of epithelium. (**C**) The inflammatory reaction, composed of mainly lymphocytes (arrow) and mast cells (arrowhead), was located mainly around vessels (V) (H&E stain).

Histologically, the swim bladders of five (cases 1#, 2#, 3#, 4#, 6#) out of the six fish sampled presented moderate to severe diffuse or perivascular inflammatory cell infiltration of the submucosa and muscularis mucosae, composed of macrophages, lymphocytes, and a few mast cells, associated with neoangiogenesis and granulation tissue (chronic aerocystitis) (Figure 1C, Figure 3D and Figure 5A–C). Four cases (cases 1#, 4#, 5#, 6#) also showed a severe thickening of the muscularis mucosae (fibrosis) (Figure 1C, Figure 2D, Figure 3D and Figure 4C), while four cases (cases 1#, 2#, 4#, 5#) showed a severe diffuse epithelial hyperplasia of the mucosa. In two of these cases (cases 2#, 4#), the hyperplasia was associated to intraepithelial eosinophilic round aggregates compatible with parasitic stages (Figure 1D). Case 2# presented also mucous metaplasia (Figure 5A,B), whereas squamous metaplasia was observed in case 1# (Figure 4C). In one case (case 5#), the epithelial hyperplasia assumed the aspect of papillary structures with intraepithelial lymphocytes and mast cells’ infiltration (inflammatory papillary hyperplasia) (Figure 2D). All the histological results are summarized in Table 1.

### 3.3. Bacteriology

Six out of seven tested swim bladder fluids resulted positive to bacteriological examinations (Table 2). Two isolates were identified by API 20 NE system (BioMèrieux, Schaerbeek, Belgium) as the A. hydrophila/caviae group (cases 2#, 4#). Molecular biological analysis performed on the other four cases (cases 1#, 7#, 8#, 9#) identified *Shewanella xiamenensis,* showing 100% nucleotide identity with strain S4 Genbank accession number NR_116732. *S. xiamenensis* 16S rDNA sequences were deposited into the GenBank database and are available under the following accession numbers: MW131343-MV131346. The antimicrobial susceptibility pattern showed a sensitivity to all tested molecules, except for one strain which was resistant to tetracycline and cyprofloxacin (Table 3).

## 4. Discussion

The swim bladder derives embryologically from the gastrointestinal tract, and it functions as a hydrostatic organ. In physostomous fish, like cyprinids, the swim bladder has a pneumatic duct that physically connects the chamber to the foregut, permitting gas to enter into or to be released through the alimentary canal (“air gulping”). Due to this particular structure, opportunistic infections related to poor water quality can occur when opportunistic bacteria enter the physostomous duct [17].

Koi carp described in this study with infectious aerocystitis (i.e., inflammation of the swim bladder) typically presented abnormal buoyancy, lethargy, and a presence of fluid inside the swim bladder easily visible through diagnostic imaging. Diagnostic imaging was useful to investigate the presence of a mass effect and to detect swim bladder lesions, also reported by other authors [2,18]. Ultrasound-guided aspiration technique [2,17,19] was useful to collect fluid from swim bladder to deepen bacteriological investigation. Bacteria such as *Aeromonas* sp., *Pseudomonas* sp., *Micrococcus* sp., and *Mycobacterium* sp. have been isolated from the swim bladders of carp with loss of neutral buoyancy and swimming anomalies [17,20]. Furthermore, descriptions of mycotic infections primarily affecting the swim bladder have been sporadically reported in cyprinids [17,21]. Other non-infectious swim bladder disorders include collapse, overinflation, displacement, deformity, and herniation, generally due to genetically based factors, breeding selection in “fancy” cyprinid varieties, stress conditions, and neoplasms [10,19,22]. Furthermore, a specific condition called swim bladder inflammation (SBI) has been reported since the 1980s as an acute and severe disease of common carp (*Cyprinus carpio*) fingerlings, associated with the presence of the myxozoan *Sphaerospora dykovae* (previously *S. renicola*) [11,12,13]. On the basis of the veterinary practitioner co-author’s clinical experience, swim bladder disorders in koi carp are being increasingly encountered. Beyond the clinical and pharmacological management of the disease, swim bladder disorders are largely underdiagnosed in koi carp and pathological investigation is rarely performed.

The present work aimed to investigate the possible etiologies and pathological findings in some cases of swim bladder disorders in adult koi carp presenting typical clinical signs. Microbiological investigation showed the presence of bacteria association with swim bladder disorders in six out of seven investigated cases (86%), including *A. hydrophila/caviae* and *S. xiamenensis*. Both primary and secondary bacterial infections of swim bladder have been reported [17]. In particular, bacteria of the genus *Aeromonas* spp. have been previously associated with aerocystitis; however, their role in the etiology of the disease has been debated [20].

*Aeromonas* sp. are a major component of intestinal microbiota in carp [23], on the other hand, *Aeromonas* sp. are generally regarded as opportunistic bacteria and isolated from diseased fish of several species, including carps [24,25,26,27]. Despite that no kidney or blood cultures were performed in our study and consequently no data are available regarding the presence of a systemic infection, a swim bladder local infection due to isolated bacteria can be assumed as this mechanism of infection has been experimentally demonstrated [20]. Indeed, inoculation of bacteria such as *Aeromonas* spp. and *Pseudomonas* spp. directly to the lumen of the swim bladder resulted in lesions identical to the ones observed in naturally occurring swim bladder disease in carp, suggesting that only infection of the swim bladder lumen, probably by way of the pneumatic duct, can cause swim bladder disease [20]. Furthermore, lesion progression was greatly affected by bacterial number and environmental conditions, with incurable outcome in case of reinfection [20]. On the other hand, lesions receded when fish were put in good environmental conditions [20], showing the importance of proper management of fish affected by swim bladder disorders. In our case series, four swim bladder anomalies (44%), such as malformation, supernumerary swim bladder, enlargement and misalignment, and a coelomic tumor (gonadal germ-cell tumor), were present and might have facilitated the bacterial entry. Regarding *Shewanella* spp., they are a heterogeneous group of microorganisms widespread mainly in marine/brackish environments [28]; moreover, these microorganisms can spread to freshwater ecosystems, being able to grow on media without NaCl [26].

*Shewanella* spp. can be found commonly in the physiological microflora of fish and have been isolated from asymptomatic fish; nevertheless, they are also reported at an increased frequency as opportunistic human and fish pathogens [28,29,30]. In particular, *S. xiamenensis* has been isolated from diseased ornamental fish [28,30]. In our study, four out of the seven cases that underwent to bacteriological analyses showed the presence of pure culture of *S. xiamenensis* from swim bladder fluid, suggesting a putative role of this pathogen in the pathogenesis of swim bladder disorder. Similarly, it was recently reported that the pure bacterial growth of the *S. putrefaciens* group from the internal organs of diseased koi carp confirmed the pathogenic ability of these microorganisms and suggested the presence of potential virulence factors involved in the pathogenetic mechanisms [28,29]. Fortunately, a wide range of sensitivity was shown to antimicrobials for isolated *S. xiamemensis* strains in accordance with other studies, where also only a few resistances were described in *Shewanella* spp. isolates [31,32]. Accordingly, antibiotic treatment succeeded in the full recovery of three cases in which *S. xiamemensis* infection was detected.

The presence of intraepithelial parasitic stages in two cases out of the six histologically investigated (33%), can also be considered a primary cause of swim bladder inflammation, which should not be underestimated, especially considering the recent literature report of swim bladder inflammation outbreaks caused by *Sphaerospora* spp., also in koi carp [11].

The histopathological changes observed in most of the sampled swim bladders were severe, thus compromising the quality of life of the animals, which were in some cases not able to recover after a medical treatment. 

## 5. Conclusions

A multidisciplinary clinical and diagnostic approach is necessary for the deepening of this condition in koi carp: diagnostic imaging is a useful noninvasive tool that supports the diagnosis of swim bladder disorders and allows a sampling of the exudate eventually present by simple echo-guided fine needle aspiration from live fish. In this way, cytological and microbiological investigations can be easily and quickly performed to drive the medical intervention and raise the chances of fish survival.

## Figures and Tables

**Table 1:** Clinical investigation, outcome, laboratory analyses, and histological findings in koi carp with swim bladder disorders.

Case #	Clinical Investigations	Outcome	Laboratory Analyses	Histological Findings
1	Ultrasonographic investigation, echo-guided needle aspiration	Euthanized	Bacteriology, histology	Submucosal and muscularis mucosae inflammation, lamina propria edema, mucosal hyperplasia with squamous metaplasia
2	Ultrasonographic investigation, echo-guided needle aspiration	Euthanized	Bacteriology, histology	Submucosal, muscularis mucosae and lamina propria inflammation, mucosal hyperplasia with mucous metaplasia and intraepithelial parasitic stages
3	Ultrasonographic investigation	Euthanized	Histology	Submucosal inflammation
4	Ultrasonographic investigation, echo-guided needle aspiration	Euthanized	Bacteriology, histology	Submucosal inflammation, muscularis mucosae fibrosis, mucosal hyperplasia with intraepithelial parasitic stages.
5	X-ray	Euthanized	Histology	Muscularis mucosae fibrosis, mucosal hyperplasia and inflammation (papillary type)
6	X-ray, ultrasonographic investigation	Euthanized	Bacteriology, histology	Submucosal and muscularis mucosae inflammation, muscularis mucosae fibrosis
7	Ultrasonographic investigation, echo-guided needle aspiration	Recovered	Bacteriology	NA
8	Ultrasonographic investigation, echo-guided needle aspiration	Recovered	Bacteriology	NA
9	Ultrasonographic investigation, echo-guided needle aspiration	Recovered	Bacteriology	NA

NA: not applicable.

**Table 2:** Results of the bacteriological investigations conducted on koi carps with swim bladder disorders.

Case #	Isolated Bacteria	Method of Identification
1	*S. xiamenensis*	16 rDNA sequencing
2	Aeromonas hydrophila/cavia group	API 20 NE
4	Aeromonas hydrophila/cavia group	API 20 NE
6	Negative	NA
7	*S. xiamenensis*	16 rDNA sequencing
8	*S. xiamenensis*	16 rDNA sequencing
9	*S. xiamenensis*	16 rDNA sequencing

NA: not applicable.

**Table 3:** Case numbers, strain names, accession numbers, and antimicrobial susceptibility of the identified *S. xiamenensis* strains.

Case #	Isolated Bacteria	Strain Name	Accession Number	Antimicrobial Susceptibility					
				Amikacin	Ciprofloxacin	Sulfamethoxazole/ Trimethoprim	Tetracycline	Ampicillin/ Sulbactam	Chloramphenicol
1	*S. xiamenensis*	402.2	MW131343	S	S	S	S	S	S
7	*S. xiamenensis*	402.3	MW131344	S	S	S	S	S	S
8	*S. xiamenensis*	402.1	MW131345	S	R	S	R	S	S
9	*S. xiamenensis*	402.4	MW131346	S	S	S	S	S	S

S: sensitive; R: resistant.
